# A functional electrical stimulation system improves knee control in crouch gait

**DOI:** 10.1007/s11832-015-0651-2

**Published:** 2015-03-19

**Authors:** Sam Khamis, Raz Martikaro, Shlomo Wientroub, Yoram Hemo, Shlomo Hayek

**Affiliations:** The Gait and Motion Analysis Laboratory, Department of Pediatric Orthopaedics, Dana Children’s Hospital, Tel Aviv Sourasky Medical Center, 6 Weizman St., 64239 Tel Aviv, Israel

**Keywords:** Cerebral palsy, Crouch gait, Functional electrical stimulation

## Abstract

**Background:**

Crouch gait is a major sagittal plane deviation in children diagnosed with cerebral palsy (CP). It is defined as a combination of excessive ankle dorsiflexion and knee and hip flexion throughout the stance phase. To the best of our knowledge, functional electrical stimulation (FES) has not been used to decrease the severity of crouch gait in CP subjects and assist in achieving lower limb extension.

**Purpose:**

To evaluate the short- and long-term effects of FES to the quadriceps muscles in preventing crouch gait and achieving ankle plantar flexion, knee and hip extension at the stance phase.

**Methods:**

An 18-year-old boy diagnosed with CP diplegia [Gross Motor Function Classification System (GMFCS) level II] was evaluated. The NESS L300^®^ Plus neuroprosthesis system provided electrical stimulation of the quadriceps muscle. A three-dimensional gait analysis was performed using an eight-camera system measuring gait kinematics and spatiotemporal parameters while the subject walked shod only, with ground reaction ankle foot orthotics (GRAFOs) and using an FES device.

**Results:**

Walking with the FES device showed an increase in the patient’s knee extension at midstance and increased knee maximal extension at the stance phase. In addition, the patient was able to ascend and descend stairs with a “step-through” pattern immediately after adjusting the FES device.

**Conclusions:**

This report suggests that FES to the quadriceps muscles may affect knee extension at stance and decrease crouch gait, depending on the adequate passive range of motion of the hip, knee extension, and plantar flexion. Further studies are needed in order to validate these results.

## Introduction

Cerebral palsy (CP) is the most common cause of upper motor neuron lesions in children, causing spasticity and muscle tendon contractures, leading to bony deformation, weakness, and loss of selective motor control. Rodda et al. and Kerr Graham classified sagittal gait patterns in spastic diplegia by providing a management algorithm based on these patterns [1, 2]. Crouch gait is a major sagittal plane deviation defined as a combination of excessive ankle dorsiflexion and knee and hip flexion throughout the stance phase. This gait pattern is commonly found in children afflicted with severe CP diplegia or quadriplegia. Crouch gait often rapidly progresses during the adolescent growth spurt. Walking aids are essential in crouch gait [3].

Several hypothesized causes for crouch gait include bony malformations causing lever arm dysfunction [4, 5], tight hip flexors [6–9], and weak muscle support [5, 10–13]. One of the most common causes of crouch gait is the overlengthening of the gastrocsoleus, producing significant weakness of the gastrocsoleus extensor mechanism of the lower limbs [3]. Crouch gait can also be a natural history of the disability [1, 3].

Multilevel orthopedic surgery has been performed to correct severe crouch gait [14, 15]. The outcomes of surgical interventions vary [13, 16]. Treatment options are very limited and dependent on conservative intervention. Strengthening the extensor moment, particularly the gluteus maximus, may help improve both hip and knee extension [13].

The use of orthotics is essential in preventing crouch gait. Orthotics, such as ground reaction ankle foot orthotics (GRAFOs), are used to enhance knee extension ability. This type of orthotic frequently leads to discomfort, painful areas, and functional disability. For the GRAFOs to be effective, the lever arm should be within normal values [17].

Functional electrical stimulation (FES), a well-known intervention, has been used for many years to facilitate muscle groups during walking. The aim of the system is to facilitate the proper muscle group at the proper timing. So an accurate synchronization between the stimulator and gait cycle is essential. FES has been mainly used to control dorsiflexors muscles and prevent drop foot at the swing phase [18, 19]. Peroneal FES is widely used due to its feasibility, simplicity, and synchronization with gait.

The aim of the present report was to evaluate the short- and long-term effects of muscle stimulation in preventing crouch gait and achieving ankle plantar flexion and knee and hip extension at the stance phase by applying FES to the quadriceps muscles. Immediate and long-term effects were assessed on a subject diagnosed with CP diplegia with a crouch gait pattern.

## Methods

### Subject and experimental protocol

We present an 18-year-old boy diagnosed with CP diplegia (Gross Motor Function Classification System (GMFCS) level II] [20], without cognitive impairment. He was considered independent according to the activities of daily living. When walking outdoors, he wore GRAFOs bilaterally for 9 years with two quadripods. Indoors, he was able to ambulate without any orthotics. For long distances, he used a wheelchair or electric scooter. His main complaints were weakness, difficulty in prolonged walking and standing, ascending and descending stairs, and dependency on the orthotics. Clinical examination exhibited bilateral hip flexion contracture (Thomas test 25°), bilateral limited knee extension of 5°, bilateral extension lag of 30°, and severe hyperpronated midfoot break, leading to excessive external foot progression. Manual muscle testing revealed severe weakness: bilateral weak hip extensors (2/5), knee extensors (4/5), gastrocsoleus (2/5). No evidence of patella alta was found.

### FES

**Fig. 1:**
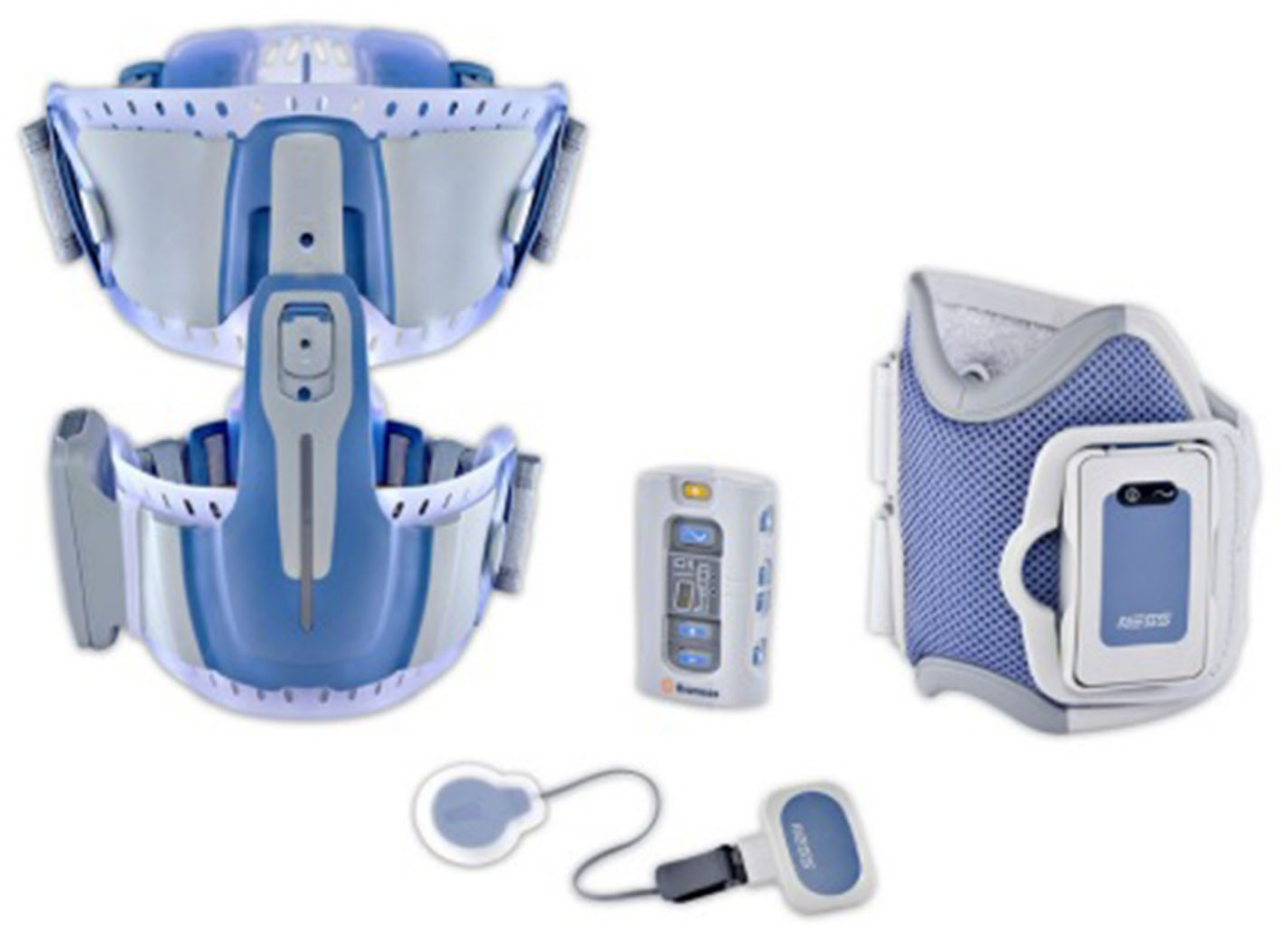
The NESS L300^®^ Plus neuroprosthesis system (NESS Ltd., Ra’anana, Israel), generating electrical stimulations to the quadriceps muscles

We used the NESS L300^®^ Plus neuroprosthesis system (Bioness Inc., Valencia, CA, USA), which delivers electrical stimulations to the common peroneal nerve, hamstrings, and quadriceps muscles (Fig. 1). For this case study, we used the NESS system for electrical stimulation of the quadriceps muscle. As described in detail by Hausdorff and Ring [21], this system has three main components that communicate via radio frequency signals: (1) a remote unit giving the patient control over the system; (2) an integrated stimulation unit with electrodes placed around the thigh (the electrodes of the thigh cuff—two oval cloth electrodes, proximal: 130 × 75 mm; distal: 120 × 63 mm—positioned over the quadriceps to extend the knee) [22]; and (3) a force-sensitive gait sensor placed beneath the foot to detect heel strike and toe off. Computed algorithms analyze the gait sensor’s data and information is then transmitted to the stimulation unit to induce knee extension at the appropriate time and exact duration [10]. Necessary adjustments may be performed by a clinician using a handheld computer (PDA) to set the stimulation intensity, pulse frequency, and gait parameters [9]. The thigh stimulation (quadriceps) can begin and end at any segment in the gait cycle, as defined by the clinician [22].

### Gait analysis system

A three-dimensional gait analysis was performed using an eight-camera system (Vicon MX Giganet motion analysis system, Oxford Metrics, UK) at 100 Hz and a capturing volume of 3.5 m–3.5 m–2.5 m. Retro-reflective markers were applied on anatomical landmarks to capture gait performance according to the biomechanical model Plug-In-Gait developed by Vicon (based on the work of Murali Kadaba and Helen Hayes Hospital) [23, 24].

### Evaluation protocol

The initial evaluation (T1) at the gait laboratory included a three-dimensional evaluation of walking at a self-selected normal speed on a 12-m walkway barefoot and wearing his own GRAFOs.

Two months after this initial evaluation (T2), he was fitted with an FES device placed on the quadriceps. During the fitting process, the stimulation parameters and timing of stimulation were set. Quadriceps stimulation was configured to start immediately after heel strike to the pre-swing phase. A symmetrical waveform was used with a phase duration of 300 μs, pulse rate 40 Hz, and intensity 40 ma. After adjusting the FES device, the subject practiced walking for 20 min prior to initiating the data capturing. After completing the fitting procedure, the subject used the system for training sessions at home on a treadmill. Every day, the subject walked 25 min and then trained for 20 min sitting. After 2 months, he walked with the FES device daily for 30 min and climbed two flights of stairs. After 6 months of conditioning (T3), he was evaluated at the gait laboratory.

At T2, he was evaluated while performing with and without the FES device (shod only). At T3, his gait was assessed in all four conditions: barefoot, GRAFOs, and with and without the FES device (shod only). Our goal was to compare the different conditions and any carryover effect on the maximum knee and hip extension and ankle plantar flexion throughout the stance phase and at midstance. Spatiotemporal parameters were compared to assess the functional effect. In addition to his walking evaluation, his ability to ascend and descend stairs was evaluated at T2 and T3.

## Results

**Fig. 2:**
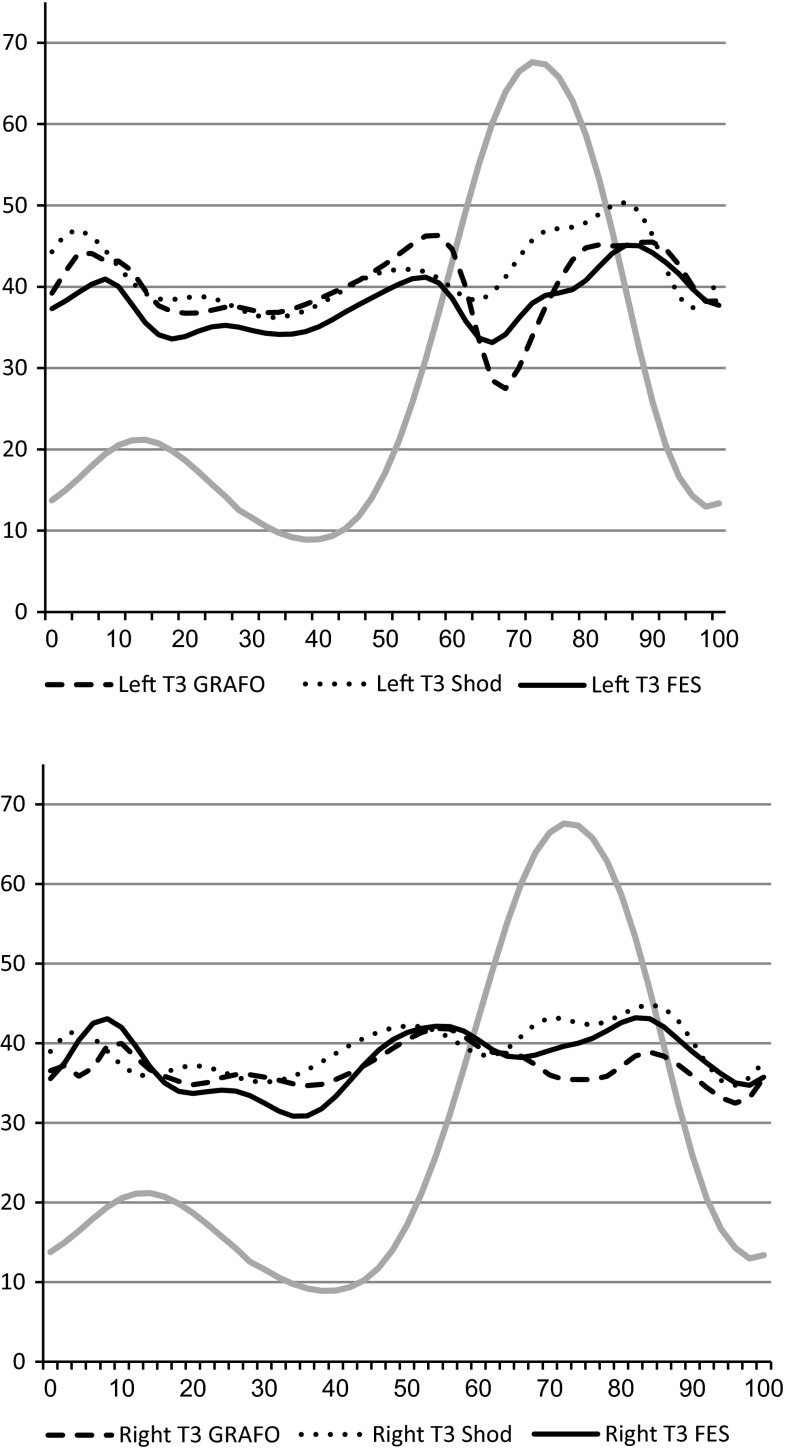
Sagittal plane movements of the left and right knee at T3 compared to typically developed (TD) subjects’ data (*gray line*). *Dotted line* shod, *dashed line* ground reaction ankle foot orthotic (GRAFO), *solid line* functional electrical stimulation (FES)

Tables 1, 2, and 3 summarize the kinematic results compared to typically developed (TD) subjects’ data recorded at the gait laboratory. Sagittal plane movement left and right knee at T3 are illustrated in Fig. 2.

**Table 1:** Sagittal plane kinematic measures of the knee at T1 [mean ± standard deviation (SD)] compared to typically developed (TD) subjects (mean ± SD)

	T1				TD
	Barefoot		GRAFO		
	Left	Right	Left	Right	
Maximal knee extension at midstance (°)	36.35 (1.29)	32.08 (1.98)	39.64 (2.40)	34.01 (1.78)	8.95 (1.89)
Maximal knee extension at the stance phase (°)	33.01 (0.08)	30.74 (0.98)	36.24 (1.83)	32.15 (1.55)	8.86 (2.15)

**Table 2:** Sagittal plane kinematic measures of the ankle and knee at T2 compared to TD subjects (mean ± SD)

	T2				TD
	Shod		FES		
	Left	Right	Left	Right	
Maximal dorsiflexion at midstance (°)	21.50 (0.95)	16.18 (0.37)	19.17 (1.95)	15.60 (0.72)	17.61 (1.22)
Maximal dorsiflexion at the stance phase (°)	31.44 (0.29)	24.43 (1.11)	28.80 (0.55)	21.74 (0.933)	19.19 (1.83)
Maximal knee extension at midstance (°)	39.16 (0.96)	37.36 (1.33)	33.23 (0.35)	36.26 (0.85)	8.95 (1.89)
Maximal knee extension at the stance phase (°)	38.63 (1.09)	37.23 (1.22)	32.36 (0.67)	34.44 (0.16)	8.86 (2.15)

**Table 3:** Sagittal plane kinematic measures of the ankle and knee at T3 compared to TD subjects (mean ± SD)

	T3						TD
	GRAFO		Shod		FES		
	Left	Right	Left	Right	Left	Right	
Maximal dorsiflexion at midstance (°)	18.55 (1.55)	15.53 (1.54)	14.05 (1.59)	16.25 (1.19)	11.18 (0.84)	15.78 (0.29)	17.61 (1.22)
Maximal dorsiflexion at the stance phase (°)	25.55 (0.38)	21.37 (0.71)	31.44 (1.58)	30.75 (0.55)	30.32 (1.20)	30.44 (0.25)	19.19 (1.83)
Maximal knee extension at midstance (°)	38.03 (1.12)	34.99 (0.67)	34.99 (0.86)	35.22 (1.44)	33.42 (1.58)	33.45 (1.50)	8.95 (1.89)
Maximal knee extension at the stance phase (°)	36.69 (1.38)	34.26 (0.45)	33.71 (0.64)	34.29 (1.47)	32.65 (1.09)	31.04 (0.75)	8.86 (2.15)

### T1 comparing barefoot to GRAFOs (Table 1)

At T1, the subject demonstrated a severe crouch gait pattern, with 36.35° left knee flexion at midstance and 32.08° on the right. Walking with GRAFOs at T1 did not improve knee kinematics. A decrease in knee extension was demonstrated at midstance and in maximum knee extension at the stance phase. Knee flexion increased by 3.29° on the left and 1.93° on the right at midstance, while maximum knee extension at the stance phase deteriorated by 3.23° on the left and 1.41° on the right. Similar results were observed at T3.

### T2 comparing shod only to FES (Table 2)

At T2, walking with the FES device showed an increase in the patient’s ankle plantar flexion and knee extension at midstance (left ankle 2.33°, right ankle 0.58°, left knee 5.93°, right knee 1.1°) and increased knee maximal extension, and ankle plantar flexion at the stance phase (left ankle 2.64°, right ankle 2.69°, left knee 6.27°, right knee 2.79°). No change occurred at the hip level during the stance phase.

### T3 comparing FES to GRAFOs and shod only conditions (Table 3)

The left knee was more extended at midstance with shoes only when compared to GRAFOs at T3 (38.03° and 34.99°, respectively). No change was found on the right. However, maximal ankle dorsiflexion increased with shoes only at the stance phase (by 5.89° on the left and 9.38° on the right), demonstrating that the GRAFOs’ maximal effect occurs at terminal stance.

Walking with the FES device at T3 revealed improvement in knee extension ability at midstance and maximal knee extension at the stance phase bilaterally compared to shoes only and GRAFOs conditions (maximum knee extension at stance, left and right): FES 32.65°, 31.04°, shod only 33.71°, 34.29°, and GRAFOs 36.69°, 34.26°. Similar results were found at T2; however, there was no effect on hip kinematics.

### Spatiotemporal parameters (Table 4)

Spatiotemporal parameters at T3 revealed an increase in walking speed with the FES device and GRAFOs compared to walking barefoot (left: 0.73, 0.67, and 0.57 m/s, respectively; right: 0.74, 0.67, and 0.55 m/s, respectively). When walking with the FES device, the highest speed was attained due to an increase in step length and cadence. When comparing the patient’s walking speed with FES in T3 versus T2, a higher walking speed, cadence, and bigger step length occurred at T3.

**Table 4:** Spatiotemporal parameters measures at T3 compared to TD subjects (mean ± SD)

	GRAFO 3		NESS off 3		NESS on 3		TD
	Right	Left	Right	Left	Right	Left	
Walking speed (m/s)	0.67 (0.03)	0.67 (0.05)	0.59 (0.03)	0.61 (0.03)	0.74 (0.02)	0.73 (0.02)	1.39
Step length (m)	0.52 (0.02)	0.52 (0.03)	0.52 (0.03)	0.5 (0.01)	0.55 (0.03)	0.56 (0.00)	0.74
Cadence (steps/min)	76.2 (2.24)	76.2 (3.16)	69.79 (1.47)	72.29 (0.44)	77.77 (1.47)	78.36 (3.46)	111

### Stairs

At T2, the patient was able to ascend and descend stairs with a “step-to” pattern, whereas he was able to ascend and descend the stairs with a “step-through” pattern immediately after adjusting the FES. This effect was also seen at T3. In addition, at T3, he was able to “step through” stairs in all conditions.

## Discussion

To the best of our knowledge, this is the first report evaluating the short- and long-term effects of an FES device on the quadriceps, compared to GRAFO, aimed to decrease the severity of crouch gait in a CP subject and assist in achieving lower limb extension. It is the first step to use rapidly developing technology to improve gait and functional ability in CP children by FES. Our patient’s gait was a typical crouch gait with excessive hip and knee flexion and dorsiflexion throughout the stance phase. He has been wearing GRAFOs for 9 years (for most of the day), together with two quadripods. However, with GRAFOs, he still complained of muscle fatigue when standing for prolonged periods of time and walking.

The subject preferred GRAFOs over barefoot and shoes only, in spite of the fact that his kinematic results did not reveal significant improvement in his lower limb extension. It was surprising to find that his knee kinematics still revealed a crouch gait pattern when comparing GRAFOs and shoes only conditions. It appears that the orthotics in this case prevented lower limb flexion rather than facilitated extension. The subject wore standard sports shoes with a higher heel than the forefoot, which might have caused forward tilting of the tibia, leading to flexion at all levels. The GRAFOs also supported him and, with proprioceptive feedback, facilitated leaning forward against the orthotics rather than knee extension.

These results concur with previous studies, where knee flexion was not reduced as a result of solid ankle foot orthosis (AFO) or GRAFOs at the stance phase. Hayek et al. [25] reported no change in ankle dorsiflexion and knee flexion angle at single limb support in children with diplegic CP using solid and articulated AFOs. Abel et al. [26] reported no significant change in knee position at stance as a result of fixed AFOs in the CP diplegia group with equinus or pes planovalgus deformities. Rethlefsen et al. [27] also reported no change in knee position during stance in subjects with spastic diplegia walking with fixed or articulated AFOs. The limited influence of orthotics on our subject with crouch gait can be explained by his severe external foot progression. Excessive external foot progression causes lever arm dysfunction, thus decreasing the efficiency of the orthotics [17]. Our results are consistent with the results of Postans and Granat [28]. They found an immediate increase in knee extension at mid terminal stance. The exact data were not reported and, thus, cannot be compared. However, they did not evaluate the long-term effects after daily use and training with an FES device nor did they compare the results to walking with shoes only or GRAFO.

Comparing all shod conditions from a kinematic point of view favored FES by improving the patient’s ability to extend the knees at the stance phase. The stimulation mainly affected the knee joint and with a small effect on the ankle. However, the hips were unaffected. The subject favored FES over GRAFOs due to achieving a more extended support on the lower limb without excessive orthotic support. In addition, FES gave him the feeling of actively strengthening and extending the knee as opposed to the passive effect of the orthotics. However, the effect of “attaining stronger muscles” as described by the subject should be further evaluated. In addition, the most impressive effect was that FES provided much more support, increased the amount of time the patient was able to walk or stand, and achieved a more upright position. This was consistent for 6 months. The positive effect can be attributed to the trend seen in Fig. 2, which shows his ability to achieve a more extended knee position throughout the stance phase. Some difficulty with the use of the FES device was reported due to the discomfort caused by sitting while the device was connected to his thigh.

It should be emphasized that FES will assist in achieving knee extension by supporting the muscle force against restraints preventing knee extension. Resistance to extension can be a short muscle–tendon or joint contractures, such as knee or hip joint flexion contracture, as is common in crouch gait. Our patient had a knee flexion contracture of 5° and tight hip flexors (Thomas test 15°). In cases of severe contractures, the FES effect might be limited.

Walking speed increased at T3, indicating that the patient acclimated to the FES device over time. The walking speed increased due to the increased step length. A carryover effect might have occurred over time on the left side only.

The effect of the stimulation on the patient’s functional ability to ascend and descend stairs was immediate, with no need for reeducation. At T2, he was able to ascend and descend stairs, “step through” versus “step to” (which had been his strategy for years), immediately after adjusting to the FES device, which was achieved by receiving more support from the stance limb and its extension, rather than leaning on the device. However, the kinematics were not captured in the stairs condition. A carryover effect with stairs occurred at T3. The subject was able to ascend and descend stairs in all conditions in a “step-through” pattern. It seems that the effect is not only due to stronger quadriceps but also due to a motor learning effect.

## Conclusions

This report suggests that functional electrical stimulation (FES) of the quadriceps muscles may have an important effect on achieving knee extension at stance and decreasing crouch gait, depending on an adequate passive range of motion of the hip, knee extension, and plantar flexion. Patient satisfaction was high due to improved functional ability and the feeling of active strengthening. The positive effect observed demonstrates the potential of FES as a means of achieving improved gait kinematics and functional ability in a patient with crouch gait. Further studies are needed in order to validate these results.
